# Multi-Patch Hierarchical Transmission Channel Image Dehazing Network Based on Dual Attention Level Feature Fusion

**DOI:** 10.3390/s23167026

**Published:** 2023-08-08

**Authors:** Wenjiao Zai, Lisha Yan

**Affiliations:** College of Engineering, Sichuan Normal University, Chengdu 610101, China; zaiwenjiao@sicnu.edu.cn

**Keywords:** transmission channels, non-homogeneous fog, dual attention, DAMPHN, image defogging

## Abstract

Unmanned Aerial Vehicle (UAV) inspection of transmission channels in mountainous areas is susceptible to non-homogeneous fog, such as up-slope fog and advection fog, which causes crucial portions of transmission lines or towers to become fuzzy or even wholly concealed. This paper presents a Dual Attention Level Feature Fusion Multi-Patch Hierarchical Network (DAMPHN) for single image defogging to address the bad quality of cross-level feature fusion in Fast Deep Multi-Patch Hierarchical Networks (FDMPHN). Compared with FDMPHN before improvement, the Peak Signal-to-Noise Ratio (PSNR) and Structural Similarity Index Measure (SSIM) of DAMPHN are increased by 0.3 dB and 0.011 on average, and the Average Processing Time (APT) of a single picture is shortened by 11%. Additionally, compared with the other three excellent defogging methods, the PSNR and SSIM values DAMPHN are increased by 1.75 dB and 0.022 on average. Then, to mimic non-homogeneous fog, we combine the single picture depth information with 3D Berlin noise to create the UAV-HAZE dataset, which is used in the field of UAV power assessment. The experiment demonstrates that DAMPHN offers excellent defogging results and is competitive in no-reference and full-reference assessment indices.

## 1. Introduction

UAVs have been increasingly employed in power inspection to find safety problems effectively [1]. However, in hilly regions, advection fog, uphill fog, and valley fog are frequently encountered [2,3], causing critical portions of transmission lines or towers to become fuzzy or even wholly concealed and decreasing fault detection accuracy. Image-defogging technology can be used to address the appeal issues. However, the non-homogenous fog is challenging for the current homogenous fog removal method. Additionally, the initial non-homogeneous defogging method FDMPHM exploits residual connections between several levels and ignores the issues with channel redundancy and unequal pixel distribution in cross-level fusion. Based on this, we suggest the Dual Attention Level Feature Fusion Multi-Patch Hierarchical Network (DAMPHN), which aims to enhance the cross-level fusion method of FDMPHN and produce superior defogging effects. Haze non-uniformity is not considered in power inspection image defogging studies due to a lack of non-homogeneous haze datasets. Therefore, to create a dataset that may represent non-homogeneous haze in mountainous places (UAV-HAZE), this paper ingeniously combines image depth measurements with 3D Berlin noise. The suggested DAMPHN performs better in color preservation and haze removal than the other four advanced approaches and can complete the picture preprocessing of transmission channels, according to numerous experiments on three open datasets and UAV-HAZE.

### 1.1. Related Work

Model-based parameter estimation and model-free picture enhancement methods are currently the main single-image fog removal categories. Additionally, future images for machine vision services will be of higher quality because of advancements in CCD imager technology [4]. Some researchers have used the image defog technique to preprocess photos based on high-quality photographs for transmission channels.

#### 1.1.1. Model-Based Parameter Estimation Method

By predicting the transmission matrix t(x) and global atmospheric light A from the haze graph J(x,λ), these approaches, based on the atmospheric scattering model [5], provide images I(x,λ) that are devoid of haze. In Equation (1), the atmospheric scattering model is displayed.

(1) $$I(x,\lambda )=t\left(x\right)J\left(x,\lambda \right)+A\left(1-t\left(x\right)\right)$$

(2) $$t\left(x\right)=e^{-\beta \left(\lambda \right)d\left(x\right)}$$

where $d\left(x\right)$ denotes the depth of the scene and $\beta \left(\lambda \right)$ the scattering coefficient. Both the early dark channel prior (DCP) [6] and the color decay prior (CAP) [7] were put out and offered concepts for further study. Convolutional neural networks (CNN) were later developed, and Cai et al. [8] used CNNs with various kernel parameters for the first time to extract the distinctive information of dark channel, color attenuation, maximum contrast, and hue disparity to solve the parameters. Li et al. [9] equalized $t\left(x\right)$ and A as a parameter based on Formula (1) and applied CNN and residual connection to get this parameter. Zhang et al. [10] used the Dense-Net and U-net networks, respectively. A Densely Connected Pyramid Dehazing Network (DCPDN) was subsequently proposed based on the joint discriminator of adversarial networks and the optimization parameter estimate of the edge retention loss function. To achieve adaptive fusion, Li et al. [11] employed a multi-stage deep convolutional network to estimate $t\left(x\right)$ and A and added a memory network and a two-level attention mechanism to determine the weight of findings at each stage. To filter haze residuals step by step and achieve dehazing, Li et al. [12] modified Formula (1) to be task-oriented and assembled recurrent neural networks based on encoder-decoder and space. Bai et al. [13], who combined $t\left(x\right)$ and A into a single parameter and calculated it using the depth pre-defamer. The progressive feature fusion module and the picture recovery module were created to improve parameter estimation.

#### 1.1.2. Model-Free Image Enhancement Method

This technique uses a coding-decoding structure to directly learn the link between the haze/clear image mapping and integrates attention mechanisms, feature fusion, and other techniques to enhance the dehazing performance. Das et al. [14] introduced the Fast Deep Multi-Patch Hierarchical Network (FDMPHN) and Fast Multi-Scale Hierarchical Network (FDMSHN) by improving the loss function, which was inspired by literature [15]. According to Wang et al. [16], a heterogeneous twin network was suggested, U-Net was used to extract haze features, and a detail enhancer network was set up to improve image details. Liu et al. [17] proposed an attention-based multi-scale defogging network (GridDehazeNet), which introduced a channel attention mechanism to improve feature fusion ability among multiple scales. A feature fusion attention network with a channel and pixel focus that prioritizes high-frequency and dense hazy areas was proposed by Qin et al. [18]. To improve the ability to extract edge texture features, Wang et al. [19] created the edge branch module based on the multi-level attention dehazing module and the feature fusion module based on Laplace gradient prior knowledge. Using extended convolution in the multi-scale part, channel attention mechanism in the cross-level fusion part, and frequency domain loss in the loss function part, Yang et al.’s [20] combination of FDMPHN and FDMSHN methods to obtain dense feature maps produced good results. A transfer attention technique was created by Wang et al. [21] to deal with non-uniform noise in images. To focus on the non-uniform hazy region and address the issues of artifacts and excessive smoothing, Zhao et al. [22] developed a dynamic attention module based on the dual attention mechanism. Guo et al. [23] suggested a self-paced half-course learning-driven attention image-generating technique based on the dual attention mechanism to enhance the ability to clear regions with considerable brightness disparities of fog.

#### 1.1.3. Transmission Channel Image Dehazing Method

Recently, researchers have used it in power inspection after taking inspiration from the appeal algorithm. Liu et al. [24] created their own UAV picture collection for transmission line inspection and used the DCPDN approach to achieve dehazing. To address the drawbacks of the DCP method, Zhang et al. [25] divided the sky region by fusing the Canny operator and gradient energy function to obtain a more accurate atmospheric light value, and Zhai et al. [26] optimized the quadtree segmentation method. Both techniques were then applied to the image dehazing of transmission line monitoring systems. To remove haze from photographs of an insulator umbrella disk in transmission lines, Xin et al. [27] coupled a limited-contrast adaptive histogram equalization method with the dark channel, bright channel, and these methods. Gao et al.’s [28] use of DCP to remove haze from fixed-point monitoring photographs of a tower or pole was likewise based on this technique. Yan et al. [29] created their dataset for UAV power inspection and used FDMPHN to achieve dehazing.

### 1.2. Motivation and Contribution

The model-based parameter estimate methods produces improved outcomes in the area of picture fog removal. However, the overall image that DCP restored is dark, and color distortion can easily happen in areas of bright light. The reduction impact is weak when the depth of field shift in the image is not visible or when there is haze, as CAP is dependent on the color saturation of the image. To maximize the fog removal effect, later researchers used CNN to estimate the parameters t(x)  and A. However, both the parameter estimation methods based on CNN [8,10,11] and the parameter estimation method after the improved atmospheric scattering model [9,12,13] are subject to artifacts, color distortion, and haze residues because of the shortcomings of the atmospheric scattering model. Although the model-free image enhancement methods are not limited by the model, it depends on the ability of the network to extract and fuse the haze features. Only residual connections are used in the multi-patch network FDMPHN for cross-level feature fusion, disregarding channel differences and pixel distribution non-uniformity. Therefore, when the non-uniform characteristics of haze or the fog area are strong, it is easy for haze residue and detail blur to appear. Later researchers enhanced the network’s capacity for feature extraction by improving the attention mechanism [17,18,19,20,21,22,23], but it was also challenging to address the issue of non-uniform fog.

In the area of fog removal in power inspection images, Refs. [24,25,26,27,28] all use a uniform haze dataset created based on an atmospheric scattering model as the foundation for their analyses, neglecting the non-uniform characteristics of haze distribution in natural settings. As a result, it is only appropriate for processing images with uniform haze distribution. It performs poorly when dealing with powerful light sources and non-uniform haze, and the image quality after recovery is also subpar. Furthermore, power inspection picture fog removal is still in the uniform haze removal stage, and it is challenging to make progress due to the relative paucity of non-uniform haze datasets [30]. Therefore, this paper suggests a Dual Attention Level Feature Fusion Multi-Patch Hierarchical Network (DAMPHN) to enhance the defogging effect of UAV inspection photos of transmission lines in mountainous terrain. This work’s key contributions can be summed up as follows:

1. It is suggested to use a Dual Attention Level Feature Fusion Multi-Patch Hierarchical Network (DAMPHN) that combines an encoder-decoder module with a Dual Attention Level Feature Fusion (DA) module. The experimental results show that the network has low color distortion and a good defogging effect.
2. DA module is proposed. DA makes use of channel attention, pixel attention, and residual connection to enhance the multi-patch layered network’s cross-level feature function strategy. The DA module has strong feature fusion capabilities, as demonstrated by numerous ablation tests.
3. By calculating picture depth information and inserting 3D Berlin noise of various frequencies, 2225 pairs of non-homogeneous haze/clear images datasets are constructed based on the actual situation. The dataset can, as closely as possible, mimic the characteristics of haze dispersal in mountainous regions. Later, it is employed to support DAMPHN training and testing, which can enhance the ability of UAV inspection photos of transmission lines in mountainous locations to remove fog.

**Figure 1:**
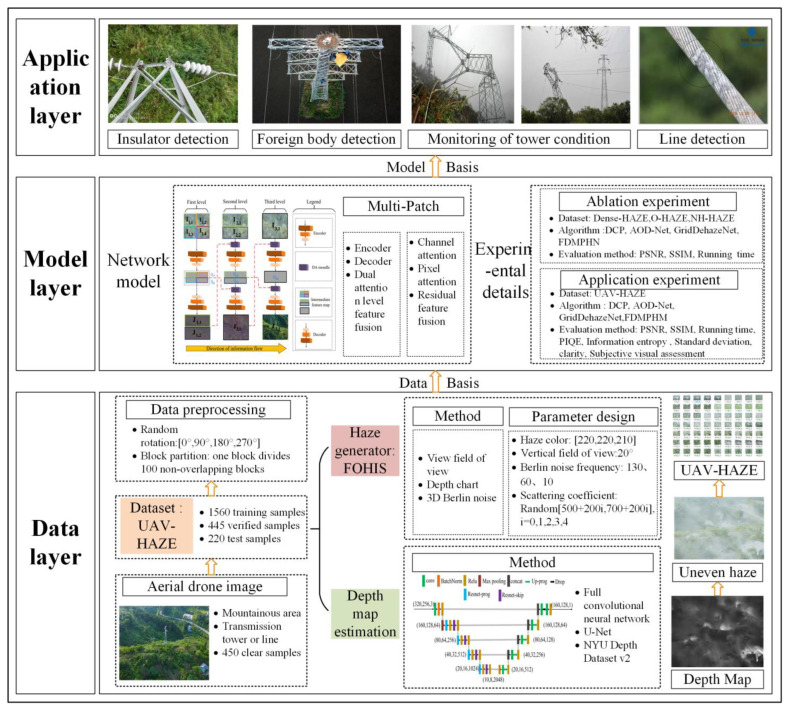
Implementation scheme of image preprocessing of mountain transmission channel based on DAMPHN.

Figure 1 illustrates the specifics of our implementation strategy for DAMPHN-based image preprocessing of mountain areas’ transmission channel images. Based on this, Section 2 details the DAMPHN network structure. It also includes the encoder-decoder and DA module’s unique construction and the loss function needed for network training. The datasets required for the ablation and application experiments and the creation of the training parameters are described in Section 3. The usefulness of the suggested DA and DAMPHN is first demonstrated in Section 4 through several ablation experiments, after which many algorithms are trained and tested using real haze photos of mountain power transmission routes and UAV-HAZE datasets. Section 5 discusses and analyzes the experimental results. In Section 6, several conclusions are made.

## 2. Materials and Methods

In this study, the encoder-decoder and DA module-based DAMPHN are suggested. This section’s first paragraph introduces DAMPHN’s architecture and design principles, as well as those of its submodules. The training and optimization of the DAMPHN loss function are covered in the second section.

### 2.1. DAMPHN

**Figure 2:**
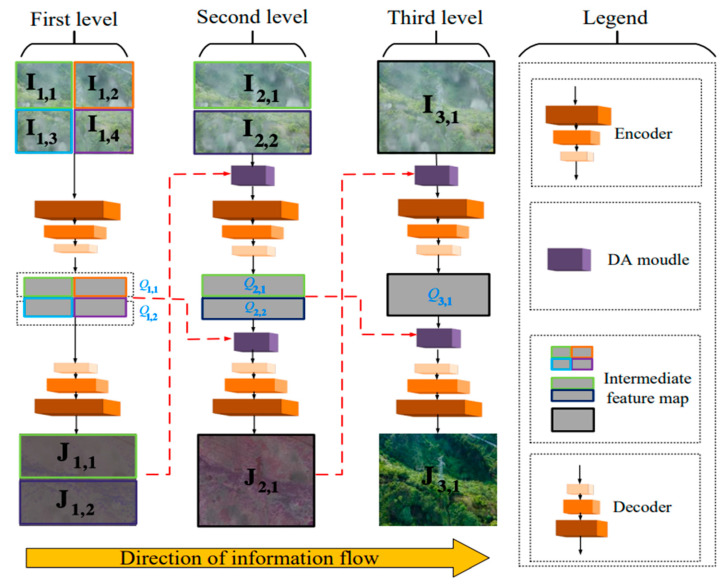
DAMPHN network structure.

DAMPHN network is a multi-level structure, and each level comprises corresponding encoders and decoders. The potential of hierarchical feature fusion is further enhanced by a Dual Attention Level Feature Fusion module (DA). Figure 2 displays the structure in its entirety. Figure 2 depicts DAMPHN with i hierarchical structure, where each level processes 4, 2, and 1 picture blocks, respectively, and when i=1,2,3. The j block of level i is represented as $I_{i,j}$ if the input image is I. The first layer then divides I into 4 blocks, identified as $I_{1,1}$, $I_{1,2}$, $I_{1,3}$, and $I_{1,4}$, both vertically and horizontally. I is divided vertically into two blocks, designated as $I_{2,1}$ and $I_{2,2}$, by the second stratum. I is directly inputted into the third layer, which is represented as $I_{3,1}$.

The pair of encoder decoders that make up each level are denoted as ${Enc}_{i}$ and ${Dec}_{i}$, respectively. The encoding feature $Q_{i,j}$ can be retrieved after the input picture $I_{i,j}$ has sequentially been through the encoder and DA module. In particular, see Equation (3).

(3) $$Q_{i,j}=\left\{\begin{array}{c} Cat[{Enc}_{i}(I_{i,2j-1}),{Enc}_{i}(I_{i,2j})],i=1,j\epsilon 1,2\\ {Enc}_{i}(DA(I_{i,j},J_{i-1,j})),i=2,j\epsilon 1,2\\ {Enc}_{i}(DA(I_{i,j},J_{i-1,j})),i=3,j=1 \end{array}\right.$$

The local feature output $J_{i,j}$ of all levels can be acquired after the DA module and decoder. $J_{3,1}$ represents the final dehazing image after DAMPHN feature extraction from the local to the overall concept. The specifics are presented in Equation (4):

(4) $$J_{i,j}=\left\{\begin{array}{c} {Dec}_{i}(Q_{i,j}),i=1,j\epsilon 1,2\\ {Dec}_{i}(DA(Cat[Q_{i,j},Q_{i,2j}],Cat[Q_{i-1,j},Q_{i-1,2j}])),i=2,j=1\\ {Dec}_{i}(DA(Q_{i,j},Cat[Q_{i-1,j},Q_{i-1,2j}])),i=3,j=1 \end{array}\right.$$

#### 2.1.1. Encoder-Decoder

**Figure 3:**
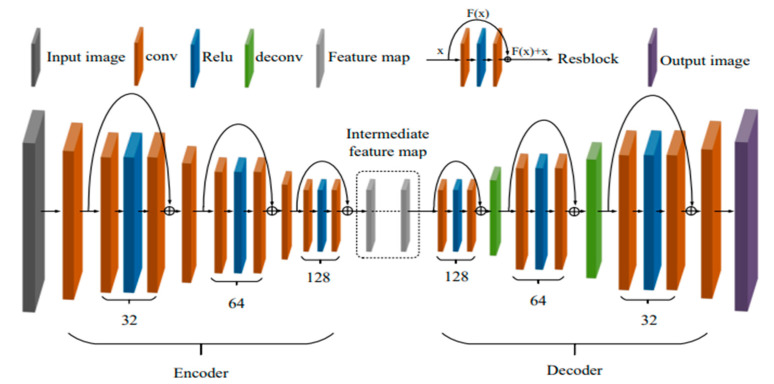
Encoder-decoder module structure.

The encoder is used to extract the feature data from the image, while the decoder reconstructs the image using the feature data. Three convolution layers and three residual modules (Resblock × 3) make up the encoder in this study. The decoder has a similar design to the encoder, with three residual modules, two transposed convolution layers, and one convolution layer. In order to generate a haze-free image and restore the image scale, decoder transposition convolution is utilized. Figure 3 depicts its network structure.

#### 2.1.2. DA Module

**Figure 4:**
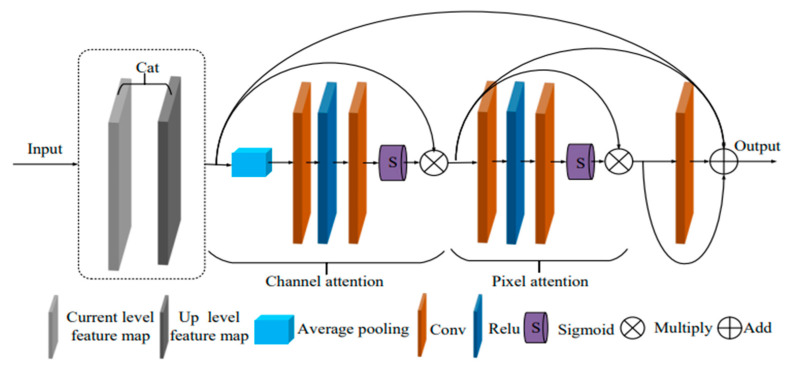
DA module structure.

After going through the encoder-decoder during the hierarchical fusion process, the local feature $J_{i,j}$ is produced from the foggy picture I input at the first and second levels. The convolution transformation of $Q_{i,j}$ yields each channel of $J_{i,j}$. As a result, the residual connection in the original FDMPHN network is employed directly in cross-level fusion, and the uneven and redundant channel direction in the fusion feature process is not considered. Additionally, the residual splicing method does not consider the uneven distribution of picture pixels, and the encode-decoder in the original FDMPHN network relies on pixel domain mapping to understand the intricate relationship between the hazy image and the clear image. This led to the development of the DA module provided in this paper, as seen in Figure 4. 

The channel domain feature response is first collected by adding the channel attention layer, and subpar or duplicated features are suppressed. Second, by including a pixel attention layer to concentrate on regions of the image with uneven pixel distribution, we may enhance the fusion process’ attention to dense haze or high-frequency regions. After stitching, input the channel attention layer (Ca_layer) and pixel attention layer (Pa_layer), assuming that the feature picture of the current level is $F_{C}\epsilon R^{H\times W\times C}$ and the feature picture of the previous level is $F_{U}\epsilon R^{H\times W\times C}$. $F_{CA}\epsilon R^{H\times W\times C}$ and $F_{PA}\epsilon R^{H\times W\times C}$ are obtained. Finally, this paper obtains the output F of the final DA module using the convolution joint processing channel and the outcomes of pixel attention processing to make up for the information lost in the extraction process of dual attention layers.

(5) $$F_{CA}=Ca\_layer(Cat[F_{C}{,F}_{U}])$$

(6) $$F_{PA}=Pa\_layer(F_{CA})$$

(7) $$F=Cat[conv(F_{PA}),F_{PA},F_{CA},Cat[F_{C}{,F}_{U}]]$$

### 2.2. Loss of DAMPHN

The total loss function L of DAMPHN is shown in Equation (8), where, respectively, $L_{r}$, $L_{p}$, and $L_{tv}$ stand for reconstruction loss, perception loss, and total variational loss.

(8) $$L=\alpha_{r}L_{r}+\alpha_{p}L_{p}+\alpha_{tv}L_{tv}$$

- Reconstruction loss $L_{r}$;

Determine the difference between the clear pictures J pixel and the N DAMPHN defogging images $J_{n}$. MAE and MSE are combined linearly. $L_{r}$ can be written as:

(9) $$L_{r}=\alpha_{r1}\frac{1}{N}\sum_{i=1}^{N}∥J_{n}-J∥+\alpha_{r2}\frac{1}{N}\sum_{i=1}^{N}∥J_{n}-J{∥}^{2}$$

- Perception loss $L_{p}$;

The VGG16 network was used to calculate features using the pre-trained model. The network’s convolution layers (Conv1-2, Conv2-2, and Conv3-2) were utilized to calculate differences, designated as φ(·), and extract features. $L_{p}$ is written as:

(10) $$L_{p}=\frac{1}{C_{K}W_{K}H_{K}}\sum_{K=1}^{3}∥\varphi_{K}(J_{n})-\varphi_{K}(J)∥$$

- Total variation loss $L_{tv}$.

$L_{tv}$ is calculated by computing the gradient amplitude of the dehazing image to reduce noise and keep the image smooth. $\nabla_{x}(\cdot )$ and $\nabla_{y}(\cdot )$ in Equation (11), respectively, are used to obtain the gradient matrix of the picture in the horizontal and vertical directions.

(11) $$L_{tv}=∥\nabla_{x}(J_{n}){∥}_{2}+∥\nabla_{y}(J){∥}_{2}$$

## 3. Experiment Setup

### 3.1. Dataset

#### 3.1.1. Ablation Experimental Dataset

The datasets for the ablation experiment were chosen from three standard datasets from the IEEE CVRP NTIRE Seminar: Dense-HAZE [31], O-HAZE [32], and NH-HAZE [33]. Dense-HAZE includes 55 identical pairs of dense haze/clear images. From the sample, 1–45 pairings were chosen for training, 46–50 pairs for verification, and 51–55 pairs for testing in this study. O-HAZE includes 45 sets of outdoor, non-homogeneous haze/clear images. From that set, 1–35 pairs were chosen for training, 36–40 pairs for verification, and 41–45 pairings for testing in this study. Fifty-five non-homogeneous haze/clear image pairs are included in NH-HAZE. In this study, 1–45 were selected for training, 46–50 for verification, and 51–55 for testing.

#### 3.1.2. Self-Built Transmission Channel Inspection Dataset (UAV-HAZE)

In haze image imaging, because it is often manifested as loss of image visibility, the atmospheric extinction coefficient σ can solve the β(λ) in Equation (12).

(12) $$\beta (\lambda )=\frac{3.912}{\sigma }$$

Additionally, visibility varies depending on height. Therefore, the depth value of the scene and the vertical field of view of the camera are used to estimate the elevation values of the pixels and their distribution characteristics are calculated to replicate the distribution and color characteristics of genuine haze. To imitate the color features of haze, Formula (1) includes the haze color value $I_{al}$ as follows:

(13) $$I(x,\lambda )=t(x)J(x,\lambda )+A(1-t(x))\times I_{al}$$

Taking into account the mountain haze’s irregularly distributed properties. Non-uniform haze is created using 3D Berlin noise, and a haze generator called FOHIS [34] is suggested. They are used to mimic non-uniform haze by making three Berlin noises of varying amplitudes and frequencies, which are then merged with Equation (13) and multiplied by β(λ).

(14) $$P\_noise=\frac{1}{3}\sum_{i=1}^{3}\frac{{P\_noise}_{i}}{2^{i}-1}$$

In light of FOHIS, this work estimated the picture depth value in order to synthesize the mountain transmission into the UAV-HAZE dataset [35]. In the synthesis process, the $I_{al}$ of the three-color channels of the image RGB is set to [220,220,210], respectively, to simulate the color characteristics of the blue-white mountain fog. Then, to imitate the distribution features of mountain haze, the vertical field of view of the camera is adjusted to 20°. This is combined with the depth value of picture pixels, and the pixel elevation value is calculated. The non-uniform properties of mountain haze were then simulated by creating 3D Berlin noise with three distinct frequency values (f = 130, 60, 10). Finally, the data [700–900], [900–1100], [1100–1300] and [1300,1500] were chosen as the extinction coefficients in Equation (12) using 450 mountain transmission channel photos obtained by UAV inspection as the original dataset. A total of 2225 non-uniform simulated haze/clear images of various concentrations make up UAV-HAZE, which is divided into training sets, verification sets, and test sets in a ratio of 7:2:1. There are 1560 pairs in the training set, 445 pairs in the verification set, and 220 teams in the test set.

### 3.2. Implementation Details

NVIDIA GeForce RTX3090 (24 GB) was the platform used for the experiment. Data preprocessing involves cropping each training image into 100 non-overlapping image blocks with a size of 120 × 160 pixels and unifying the image resolution of the training set across Dense-HAZE, O-HAZE, NH-HAZY, and UAV-HAZE to 1200 × 1600. The image blocks were simultaneously rotated at random angles of 0, 90, 180, and 270 degrees. The Adam optimizer is initially employed in DAMPHN network training with exponential decay rates $\gamma_{1}$ = 0.9, $\gamma_{2}$ = 0.999, starting learning rates 1 × 10^−4^, and batch sizes 100. We also adjusted the learning rate using an equally spaced strategy with step size = 10 and gamma = 0.1. Then, the hyperparameters of the loss function are set to $\alpha_{r}$ = 1, $\alpha_{p}$ = 6 × 10^−3^, $\alpha_{tv}$ = 2 × 10^−8^, $\alpha_{r1}$ = 0.6, $\alpha_{r2}$ = 0.4. Finally, when the verification set loss function is stable, the training is stopped and the best model is obtained.

## 4. Experiment Results

### 4.1. Ablation Experiment

Two phases of the ablation experiment were conducted. The first and second sections, respectively, confirm the reliability of the DA module and the DAMPHN network.

#### 4.1.1. DA Module

Due to the low cross-level fusion quality of the original multi-patch algorithm FDMPHN, the DA module is proposed in this study. In order to reduce the complexity of the algorithm, the encoder-decoder structure of FDMPHN is diminished. The three sets of experiments listed below are explicitly included in this section:

(I) The network encoder-decoder has six residual modules (Resblock × 6) using only FDMPHN.
(II) The approach suggested in this work builds on (I) by adding a DA module (FDMPHN + DA). A DA module plus six residual modules (Resblock × 6) make up the network encoder-decoder.
(III) To optimize (II) and DAMPHN, the solution presented in this research uses just three residual modules (Resblock × 3).

- Quantitative evaluation

PSNR [36], SSIM [37], and APT were chosen for quantitative evaluation in this section of the experiment. The visual noise and distortion decrease as the PSNR value rises. The recovery of structural properties such as image brightness and contrast is measured by SSIM. The dehazing is better the higher the value. Table 1 displays the precise outcomes of the three groups of studies. In Table 1, when (I) and (II) are compared, the addition of the DA module raised PSNR and SSIM in the three datasets by an average of 0.35 dB and 0.0073, whereas APT rose by 19% (0.007 s). Comparing (I) and (III), the average PSNR and SSIM in the three datasets are raised by 0.30 dB and 0.011, respectively, and APT is shortened by 11% (0.003 s), respectively, after the encode-decoder structure is streamlined.

- Convergence analysis

**Figure 5:**
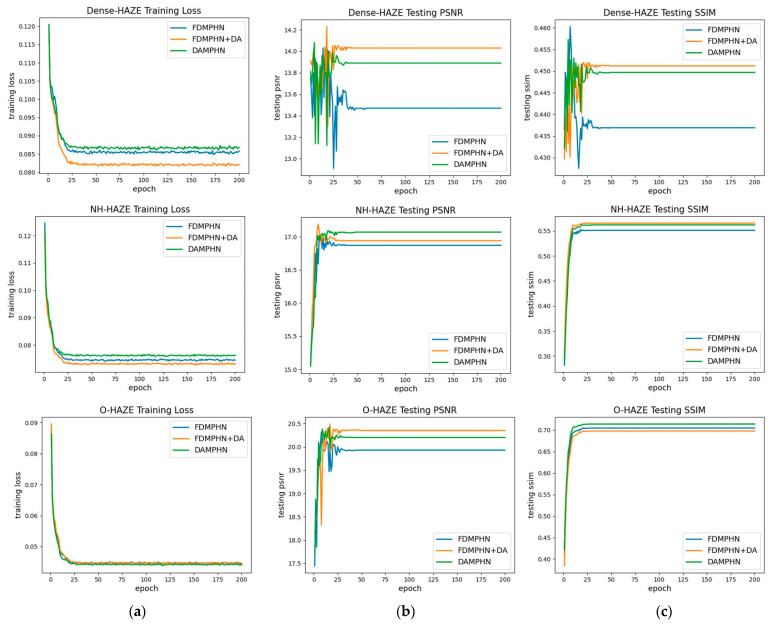
Training loss curve and test PSNR and SSIM curve. (**a**) Training loss. (**b**) Testing PSNR. (**c**) Testing SSIM.

This section assessed the convergence using the dynamic curves for training loss, PSNR, and SSIM. On Dense-HAZE, O-HAZE, and NH-HAZE, Figure 5 displays the training losses, PSNR, and SSIM for the FDMPHN, FDMPHN+DA, and DAMPHN approaches, respectively. Figure 5 shows the training and testing of the three approaches on three separate datasets, with the training losses, PSNR, and SSIM information displayed in the rows and columns, respectively. Figure 5a illustrates how the training loss for the aforementioned approaches steadily lowers as the number of iterations increases and gradually stabilizes at 35–40 rounds. In Figure 5b,c, all three approaches converge after 200 rounds, and the DA module performs better regardless of how complicated or straightforward the encoder-decoder structure is.

#### 4.1.2. DAMPHN Network 

To more accurately evaluate DAMPHN, we further conducted quantitative, qualitative, and convergence evaluation on three datasets, Dense-HAZE, O-HAZE, and NH-HAZE, with DCP [6], AOD-Net [9], FDMPHN [14], and GridDehazeNet [17], respectively.

- Quantitative evaluation

PSNR, SSIM, and APT are also used to gauge how well various techniques remove haze. The outcomes of the quantitative comparison are displayed in Table 2. In Table 2, the blue values represent the optimal values, and the underlined values represent the sub-optimal values. In the three datasets, the PSNR and SSIM values of DAMPHN are 3.72 dB and 0.0666 higher than those of DCP on average, and ART is 94% shorter. The defog quality of AOD-Net in the Dense-HAZE dataset is comparable to that of DAMPHN. However, on the non-uniform haze datasets O-HAZE and NH-HAZE, the PSNR and SSIM values of DAMPHN are increased by 1.72 dB and 0.0446 compared with the average value of AOD-Net. The effect of GridDehazeNet on the fog removal in the three datasets has its own advantages compared with the method in this paper. Specifically, DAMPHN is, on average, 0.38 dB higher than GridDehazeNet’s PSNR value, but the SSIM value is lower than GridDehazeNet’s 0.025. Finally, compared with FDMPHN in the three datasets, the PSNR and SSIM values of DAMPHN are increased by 0.30 dB and 0.011 on average, and ART is shortened by 11%.

- Qualitative assessment

**Figure 6:**
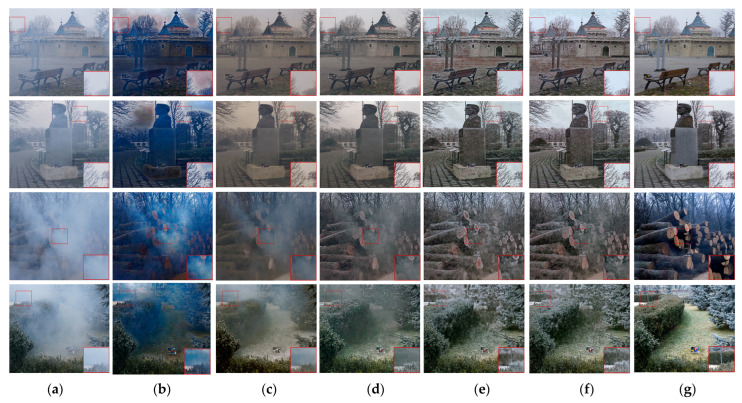
NH-HAZE and O-HAZE dehazing results. (**a**) Hazy. (**b**) DCP. (**c**) AOD-Net. (**d**) GridDehazeNet. (**e**) FDMPHN. (**f**) DAMPHN. (**g**) Ground truth.

The experiment’s visual comparison component is the main focus here. Among the images, the haze distribution in the first and second rows is more uniform, and the haze distribution in the third and fourth rows is uneven. The DCP results in Figure 6 reveal color distortion and a significant degree of residual haze. The image’s color changes to dark yellow after AOD-Net fog removal, and a significant quantity of haze residue remains in the non-uniform haze area. GridDehazeNet has a good fog effect when the haze distribution is relatively uniform, but the image’s color after fog removal is darker than that of the clear picture. In addition, in the case of non-uniform haze, GridDehazeNet also shows many haze residues. The image’s overall color after fog removal by FDMPHN is closer to the clear image when the haze distribution is more uniform. Still, the color distortion appears on the ground of the first line of the picture. Regarding non-uniform haze, FDMPHN has a good de-fogging effect, but its de-noising solid ability also causes image smoothing, resulting in blurred details. DAMPHN is visually similar to FDMPHN. However, in the enlarged area of the fourth row of the image, the DAMPHN haze residue is less.

- Convergence analysis

**Figure 7:**
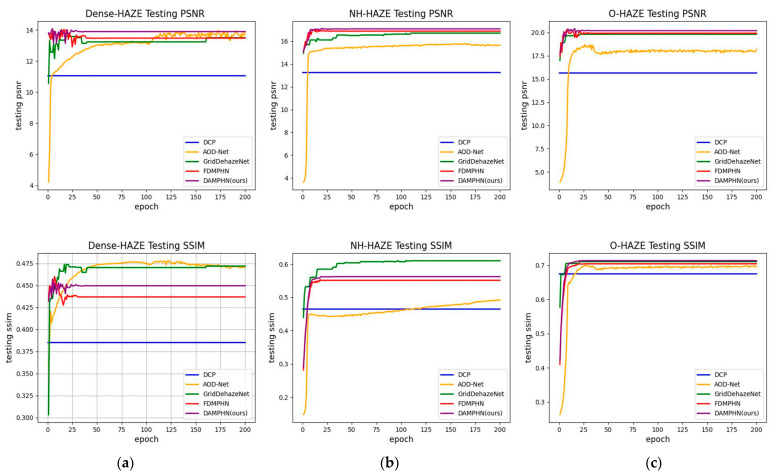
PSNR and SSIM test curves. (**a**) Dense-HAZE. (**b**) NH-HAZE. (**c**) O-HAZE.

In this experiment section, the convergence is assessed using the change curves of PSNR and SSIM with the number of training rounds. Figure 7 shows the results of each round of PSNR and SSIM tests for four de-fogging techniques on three datasets. DCP has the fastest convergence rate. AOD-Net uses a relatively lightweight CNN structure in the parameter estimation process, which has poor stability and the slowest convergence rate. When the PSNR value of the current verification set is assumed to be greater than the previous results during GridDehazeNet training, the round model is optimal. Under dynamic control, its convergence rate ranks fourth. The FDMPHN and DAMPHN set the hyperparameters before training, and the validation set is used to optimize the hyperparameter settings. Therefore, both FDMPHN and DAMPHN converge faster. Specifically, in Figure 7a, DAMPHN converges faster than FDMPHN. In Figure 7b,c, FDMPHN and DAMPHN converge at similar speeds. Therefore, DAMPHN in this paper is in second place in terms of convergence speed.

### 4.2. Transmission Channel Image 

#### 4.2.1. Synthetic Dataset UAV-HAZE

DAMPHN can be utilized to clear haze from Sichuan’s mountainous areas’ transmission channel scenery. This section is based on the dataset created in Section 3.1.2, UAV-HAZE. With this collection of data, DCP [6], AOD-Net [9], FDMPHN [14], GridDehazeNet [17], and DAMPHN, the approach in this article, are each examined in turn. This section evaluates both the algorithm’s quantitative and qualitative performance.

- Quantitative evaluation

PSNR, SSIM, and APT were chosen as evaluation indicators. Table 3 presents the experimental outcomes. In Table 3, the blue font is the optimal value, and the underlined value is the sub-optimal value. The PSNR of DAMPHN is optimal, SSIM and ART are sub-optimal. In this study, DAMPHN’s PSNR and SSIM values are 7.26 dB and 0.0588 greater than DCP’s, respectively. APT barely makes up 4% of DCP techniques. PSNR and SSIM are 9.32 dB and 0.2057 greater in DAMPHN than in AOD-Net, although APT is 14 times higher. The PSNR value of DAMPHN is 0.26 dB higher, and the SSIM value is 0.0007 dB lower than GridDehazeNet. DAMPHN’s SSIM value is the same as FDMPHN’s, but its PSNR is 0.04 dB higher, and its APT is 94% shorter.

- Qualitative assessment

**Figure 8:**
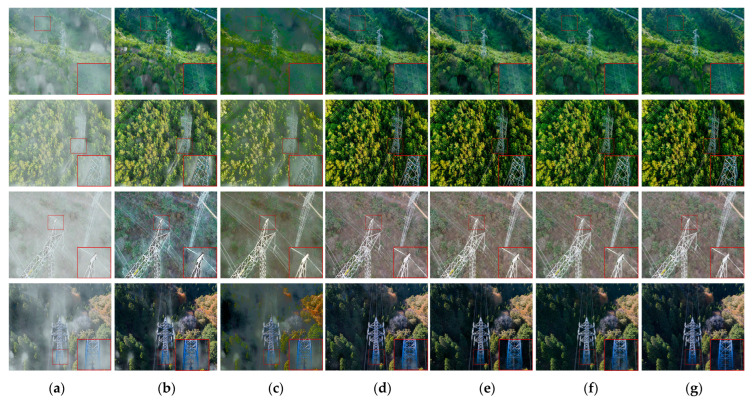
Results of UAV-HAZE dehazing. (**a**) Hazy. (**b**) DCP. (**c**) AOD-Net. (**d**) GridDehazeNet. (**e**) FDMPHN. (**f**) DAMPHN. (**g**) Ground truth.

Figure 8 displays the outcomes of the qualitative comparison between DAMPHN and the techniques mentioned above. DCP has a positive impact in the mist area, according to the analysis of Figure 8. The color of the third row seems distorted when the haze density is excellent, or the randomness of its distribution features is substantial. When dealing with non-uniform haze, AOD-Net’s primary result is that a significant amount of haze is left in the processed image, the details are blurred, and there is evident color distortion. The fog removal quality of GridDehazeNet is superior to that of the first two techniques. However, some fog was still present close to the first row’s wires and the fourth row’s poles and towers. In this study, the FDMPHN and DAMPHN techniques can recover the picture tower’s detailed information with excellent clarity and superb color fidelity. FDMPHN does, however, have a trace amount of haze residue in the first row’s wire area.

#### 4.2.2. Real Image

The actual utility of DAMPHN was confirmed by the refit project from Gangu to Erlang Mountain in Shuzhou and the real hazy photographs of the Sichuan-Tibet network project. The approach was evaluated using both quantitative and qualitative methodologies.

- Quantitative evaluation

Five non-reference image quality evaluation indexes, including information entropy, standard deviation, clarity, perception-based image quality evaluation method (PIQE) [38], and APT, were chosen for quantitative evaluation because there were insufficient clear reference examples. The more relevant information an image carries, the higher its information entropy. The image’s standard deviation is used to assess its contrast; the lower the standard deviation, the more stable the image is. The greater the value, the higher the sharpness, which is defined as the variance of calculating the absolute value of Laplace. Block effects, blur, and noise distortion are calculated using PIQE, and a lower value corresponds to less distortion. In Table 4, the experimental findings are displayed.

In Table 4, the underlined value and the blue text represent the ideal and sub-optimal values, respectively. This approach performs the best regarding clarity and PIQE, comes in second for ART, and comes in third for information entropy and standard deviation. This approach has reduced standard deviation and higher assessment indices compared to DCP. The proposed method has a clear benefit over AOD-Net regarding image quality, but it takes four times as long to operate. DAMPHN has higher evaluation indexes than GridDehazeNet, except for lower information entropy. DAMPHN is superior to FDMPHN in various assessment indices compared to FDMPHN before improvement, except for the picture information entropy, which is less than 0.17.

- Qualitative assessment

**Figure 9:**
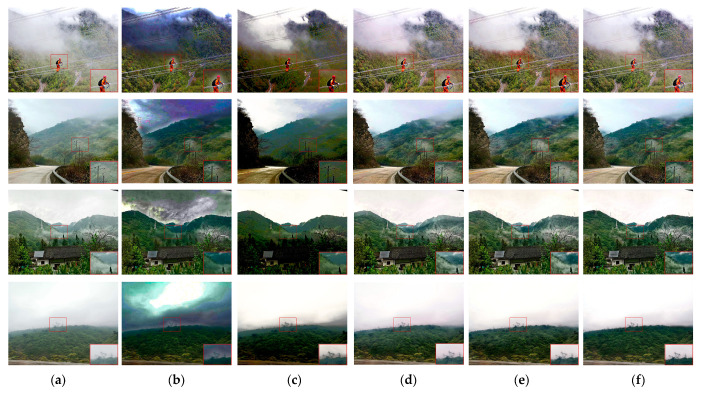
Dehazing result of real transmission channel image. (**a**) Hazy. (**b**) DCP. (**c**) AOD-Net. (**d**) GridDehazeNet. (**e**) FDMPHN. (**f**) DAMPHN.

Figure 9 displays two transmission channel views of the retrofitting project from Gangu to Erlang Mountain in Shuzhou and the haze reduction effect of four groups of the Sichuan-Tibet interconnection project. Uphill fog, uphill fog, advection fog, and radiation fog are all depicted in lines 1 through 4. Intuitive examination reveals that the color of DCP is severely altered and turns blue-purple in the sky area. AOD-Net effectively removes haze. However, it has glaring issues with blurred details and intensified hue. Although GridDehazeNet effectively removes fog, there is still some fog in the third-row valley and second-row tower areas. The image is also slightly lavender once the fog has been eliminated, for instance, the first row’s valley fog area and the fourth row’s pole tower area. In places with high haze density, such as the tower area in the second row and the valley area in the third row, FDMPHN has a competitive dehazing impact but leaves haze residue behind. This technique also results in color distortion, as seen in how the first row of trees on an ascent turned yellow. After adding a DA module, DAMPHN may now pay closer attention to areas with dense fog and a non-uniform haze. As a result, the method suggested in this paper removes fog more thoroughly than GridDehazeNet and FDMPHN in the first-row and third-row valley areas. Additionally, there is no purple or yellowing in terms of color preservation.

## 5. Discussion

In this paper, the issue of transmission line haze that is unevenly dispersed in mountainous places was studied. A DAMPHN is introduced, an innovative non-uniform haze-defogging network model put forth in this research to facilitate picture preprocessing for UAV transmission channel inspection in mountainous terrain. Similarly, the DAMPHN network model is universal. DAMPHN can be used for preprocessing other images in fog environments, such as unmanned visual perception, surveillance video (road traffic, transmission lines), and tachographs. DCP, AOD-Net, GridDenzeNet, and FDMPHN were utilized in numerous tests using open datasets (Dense-HAZE, O-HAZE, and NH-HAZE) and self-built datasets (UAV-HAZE) to demonstrate the efficacy of DAMPHN.

Notably, because the assumption of uniform distribution of air concentration in the atmospheric scattering model limits both DCP and AOD-Net, the error of estimating parameters is significant in dense fog and non-homogeneous haze. DAMPHN is a multi-level end-to-end fog removal network that seeks to remove fog by discovering the relationship between the haze and clear image mapping. DAMPHN does not, therefore, need to estimate the parameters; instead, it relies on the dataset’s basis, and the higher the base, the higher the quality of fog removal. GridDehazeNet solves the problem of feature fusion between different scales in multi-scale networks by introducing channel attention. DAMPHN solves the problem of feature fusion between different levels in multi-patch networks by introducing channel and pixel attention mechanisms. GridDehazeNet has vital artifact removal, so the SSIM value is stronger than DAMPHN. DAMPHN pays attention to the problem of uneven pixel distribution, pays attention to the removal of non-uniform fog, and has a strong denoising ability and high PSNR value. FDMPHN is identical to a multi-patch defogging network, but the residual connections in hierarchical fusion restrict how well it can fuse features. The pixel attention layer of the DAMPHN’s DA module is designed to pay attention to areas with unequal haze distribution. In contrast, the channel attention layer is designed to appropriately evaluate the channel domain properties. DAMPHN has a better defogging impact as a result than FDMPHN.

Additionally, the frequently used image segmentation algorithms U-Net and GridNet have produced effective outcomes in image segmentation and picture defogging via innovation. DCPDN solves parameter A using the U-Net network. GridDehazeNet proposes a multi-scale attention network based on GridNet. They both have superior defogging effects. With dual U-Net, Amyar et al. [39] created a multi-task and multi-scale network structure that was effectively used for lung tumor segmentation, classification, and prediction. However, DAMPHN accomplishes picture fog removal from the local to the global by helping the feature extraction of the bigger patch image from the top layer with the detailed feature of the lower layer. From the overall to the local picture segmentation, image fog removal, and other tasks, U-Net will employ the more comprehensive information collected from the bottom layer to aid in the development of smaller receptive field information. Consequently, the two networks’ designs have produced successful outcomes in their respective domains.

In conclusion, the DAMPHN approach offers an excellent defogging effect, less color distortion, and quick processing speed. In a location with a lot of fog, it is impossible to eliminate it entirely, and the details are hazy. DAMPHN can improve the defog effect by enhancing the encoder-decoder structure, feature extraction, and reconstruction skills, all of which were influenced by U-Net in the field of image segmentation, or by combining with the conventional image edge previous knowledge to increase the texture information and boost the fog removal effect.

## 6. Conclusions

This paper proposes that DAMPHN can achieve a good defog effect and restore the color and brightness of the image. The network encoder-decoder module and DA module are composed. The former can learn the mapping relationship between haze and clear pictures and has a strong feature extraction ability. The latter enhances the feature fusion ability by empowering the combination of channel attention and pixel attention. However, in excessive haze density, it cannot be entirely removed, and the details are hazy. Future work will improve the haze removal effect by enhancing texture information through edge prior and enhancing the encoder-decoder structure. Additionally, using 3D Berlin noise and image depth information to simulate haze’s non-uniform distribution characteristics is not only just restricted to UAV mountain transmission channel inspection; it can also be applied to a broader range of situations to enhance generalization performance.

## Figures and Tables

**Table 1:** Results of DA module ablation experiments.

Method		Dense-HAZE			O-HAZE			NH-HAZE		
		PSNR	SSIM	APT	PSNR	SSIM	APT	PSNR	SSIM	APT
(I)	FDMPHN	13.47	0.4369	0.031	19.93	0.7045	0.030	16.87	0.5512	0.030
(II)	FDMPHN + DA	14.03	0.4512	0.036	20.35	0.6976	0.035	16.94	0.5656	0.037
(III)	DAMPHN	13.89	0.4497	0.027	20.20	0.7138	0.027	17.07	0.5621	0.027

**Table 2:** Results of DAMPHN Network quantitative comparison.

Method	Dense-HAZE			O-HAZE			NH-HAZE		
	PSNR	SSIM	APT	PSNR	SSIM	APT	PSNR	SSIM	APT
DCP [6]	11.60	0.3854	0.406	15.66	0.6753	0.440	13.28	0.4650	0.416
AOD-Net [9]	13.85	0.4714	0.023	18.19	0.6950	0.010	15.64	0.4918	0.009
GridDehazeNet [17]	13.50	0.4721	0.026	19.82	0.7108	0.026	16.70	0.6101	0.026
FDMPHN [14]	13.47	0.4369	0.031	19.93	0.7045	0.030	16.87	0.5512	0.030
DAMPHN (ours)	13.89	0.4497	0.027	20.20	0.7138	0.027	17.07	0.5621	0.027

**Table 3:** Quantitative comparison results on UAV-HAZE.

Method	PSNR	SSIM	APT
DCP [6]	19.97	0.8851	0.352
AOD-Net [9]	17.92	0.7382	0.001
GridDehazeNet [17]	26.98	0.9476	0.015
FDMPHN [14]	27.20	0.9439	0.234
DAMPHN (ours)	27.24	0.9439	0.014

**Table 4:** Results of quantitative evaluation of real images.

Method	Information Entropy	Standard Deviation	Clarity	PIQE	APT
DCP [6]	17.78	32.19	459.86	27.51	0.342
AOD-Net [9]	16.10	45.93	452.79	28.87	0.005
GridDehazeNet [17]	18.28	41.61	470.18	24.90	0.021
FDMPHN [14]	18.10	42.38	465.21	24.48	0.270
DAMPHN (ours)	17.93	41.92	536.11	23.98	0.020

## Data Availability

Not applicable.

## Abbreviations

The following abbreviations are used in this manuscript:

UAV	Unmanned Aerial Vehicle
FDMPHN	Fast Deep Multi-Patch Hierarchical Network
DAMPHN	Dual Attention Level Feature Fusion Multi-Patch Hierarchical Network
PSNR	Peak Signal-to-Noise Ratio
SSIM	Structural Similarity Index Measure
APT	Average Processing Time
DCP	Dark Channel Prior
CAP	Color Decay Prior
CNN	Convolutional neural networks
AOD-Net	All-in-One Dehazing Network
DCPDN	Densely Connected Pyramid Dehazing Network
FDMSHN	Fast Deep Multi-Scale Hierarchical Network
DA	Dual Attention Level Feature Fusion
Ca_layer	Channel attention layer
Pa_layer	Pixel attention layer
PIQE	Perception-based Image Quality Evaluation
